# Exploring Healthcare Workers’ Knowledge, Attitudes and Behaviors to Antibiotic Use, Including in Acne, and Antibiotic Resistance in English Correctional Facilities in 2024

**DOI:** 10.3390/antibiotics15090911

**Published:** 2026-09-16

**Authors:** Clare Oliver-Williams, Chantal Edge, Denise Farmer, Diane Ashiru-Oredope, Naomi Fleming

**Affiliations:** 1 West Northamptonshire Council, One Angel Square, Angel Street, Northampton NN1 1ED, UK; 2 UK Health Security Agency, 10 South Colonnade, Canary Wharf, London E14 4PU, UK; chantal.edge@ukhsa.gov.uk (C.E.); diane.ashiru-oredope@ukhsa.gov.uk (D.A.-O.); 3 NHS England, Wellington House, 133-155 Waterloo Road, London SE1 8UG, UK; 4 NHS England, Victoria House, Capital Park, Fulbourn, Cambridge CB21 5BQ, UK

**Keywords:** antimicrobial resistance, correctional facilities, pharmacists

## Abstract

**Background:** Antimicrobial resistance (AMR) is a global health threat, driven in part by inappropriate antibiotic use. Vulnerable populations, such as individuals within the criminal justice system, potentially face heightened AMR risks due to infection control challenges and health inequalities. This study explored health and justice healthcare professionals’ views of AMR and antibiotic prescribing to identify challenges and opportunities for action. **Methods:** A quantitative survey among healthcare professionals in English correctional facilities assessed knowledge, attitudes, and behaviors towards AMR and prescribing, including acne prescribing. The survey was structured using COM-B and included 82 Likert-scale statements and 7 key AMR knowledge questions. **Results:** There were 24 respondents; 54.2% were pharmacy professionals. Both pharmacy and non-pharmacy professionals demonstrated strong AMR understanding, with 5/7 knowledge questions answered correctly by everyone. Motivation-related responses were generally positive, with the median response corresponding to strong agreement for five of six questions. Although opportunity and capability responses were generally positive, they indicated several areas where respondents reported lower levels of confidence and knowledge, such as having the opportunity to run searches on the clinical system to monitor antibiotic prescribing. Overall, 79% of respondents were able to answer all seven key AMR knowledge questions correctly. **Conclusions:** This study provides preliminary insights into prescribing behavior in health and justice settings, emphasizing the need for further research to understand health and justice healthcare workers and develop appropriate interventions to reduce AMR.

## 1. Introduction

Antimicrobial resistance (AMR) is one of the world’s largest global health challenges [1,2,3]. It is fueled by inappropriate antibiotic use, which contributes to the development of resistance in many micro-organisms which cause disease in both humans and animals [1,2]. The consequences of this are severe. Many common infections and clinical procedures will become increasingly risky, leading to greater morbidity and mortality for patients.

This may be experienced most acutely for groups of individuals who are already marginalized and vulnerable [4]. This includes residents of correctional facilities. In this review, the term correctional facilities is used to refer to prisons or other penal institutions where individuals are held pretrial and after conviction. It is already known that people in correctional facilities are at increased risk of AMR infections [5,6], and the nature of life in these facilities makes infection prevention and control and access to appropriate treatment more challenging than for those living in the community [7].

These challenges can lead to inappropriate prescribing. Acne prescribing, in particular, has been identified as a challenge and was, therefore, included as an exploratory area of interest within this study. The percentage of antibiotic prescriptions that were for lymecycline, mainly prescribed for acne, was four-times higher in English correctional facilities than in primary care in England. This suggests that acne should be a focus for antimicrobial stewardship (AMS) quality improvement.

The UK government’s 20-year vision for AMR aims to ensure AMR will be controlled and contained by 2040 [8]. To accomplish this, effective infection prevention and control are paramount as well as raising awareness of AMR among the healthcare workforce. Tackling health inequalities is also emphasized in the UK government’s 5-year (2024–2029) action plan for AMR [9] to ensure that future interventions have the greatest impact for those with the greatest need. This includes those living in correctional facilities.

The COM-B model can be used to clarify a ’problem’ (such as AMR) in behavioral terms, and it considers three key constructs, capability, opportunity and motivation, which all influence behavior. This enables an assessment of the determinants of behavior and can go on to be used to aid the development of appropriately targeted interventions. This study uses the COM-B model to explore health and justice healthcare professionals’ views of AMR and prescribing behavior, including acne management and antibiotic use, in an under-researched setting [7]. The aim was to generate descriptive evidence regarding knowledge, attitudes and behaviors related to AMS in English correctional facilities and identify areas for future investigation and intervention development.

## 2. Results

There were 41 initiations of the survey and 24 completions (58.5% completion rate). Only surveys that were completed were included in the analysis.

Table 1 summarizes the demographics of the respondents. Pharmacy professionals comprised 54.2% of respondents, with other professionals being doctors, nurses and allied health professionals. Respondents were from all English regions, and slightly more women than men responded (62.5%). Non-pharmacy professionals had worked in healthcare for longer than pharmacy professionals.

Due to limited demographic data on correctional healthcare staff in England, it is not possible to assess how representative the sample is.

Most respondents (18/24, 75%) had received information about avoiding unnecessary prescribing, administering or dispensing of antibiotics in the previous 12 months. Eight respondents (44.4%) did not change their practice based on the information they received. Another eight respondents (44.4%) did change their practice based on the information they received, and two (11.1%) were unsure.

All eight respondents who did not change their practice reported that their practice already aligned with the information they received. Among the eight respondents who did make changes, the information that was reported to be influential in changing practice was as follows: information about shorter course duration (“The shorter the better information, therefore we try to prescribe more 5 day courses if appropriate”) (four respondents), including on the FutureNHS platform (“The resources on the FutureNHS platform that I have used to more confidently recommend shorter courses of antibiotics.”); general information about the need to be more responsible, including the costs of managing resistance (one respondent); the adverse effects of antibiotics (“Information about the adverse effects of certain antibiotics and the need to ensure they are being used only when needed because other alternatives have not been effective or unsuitable”) (one respondent), including risk alerts for fluoroquinolones (1 respondent); and participation in the CQUIN IV to Oral switch (one respondent).

### 2.1. Capability, Opportunity and Motivation Questions

**Figure 1:**
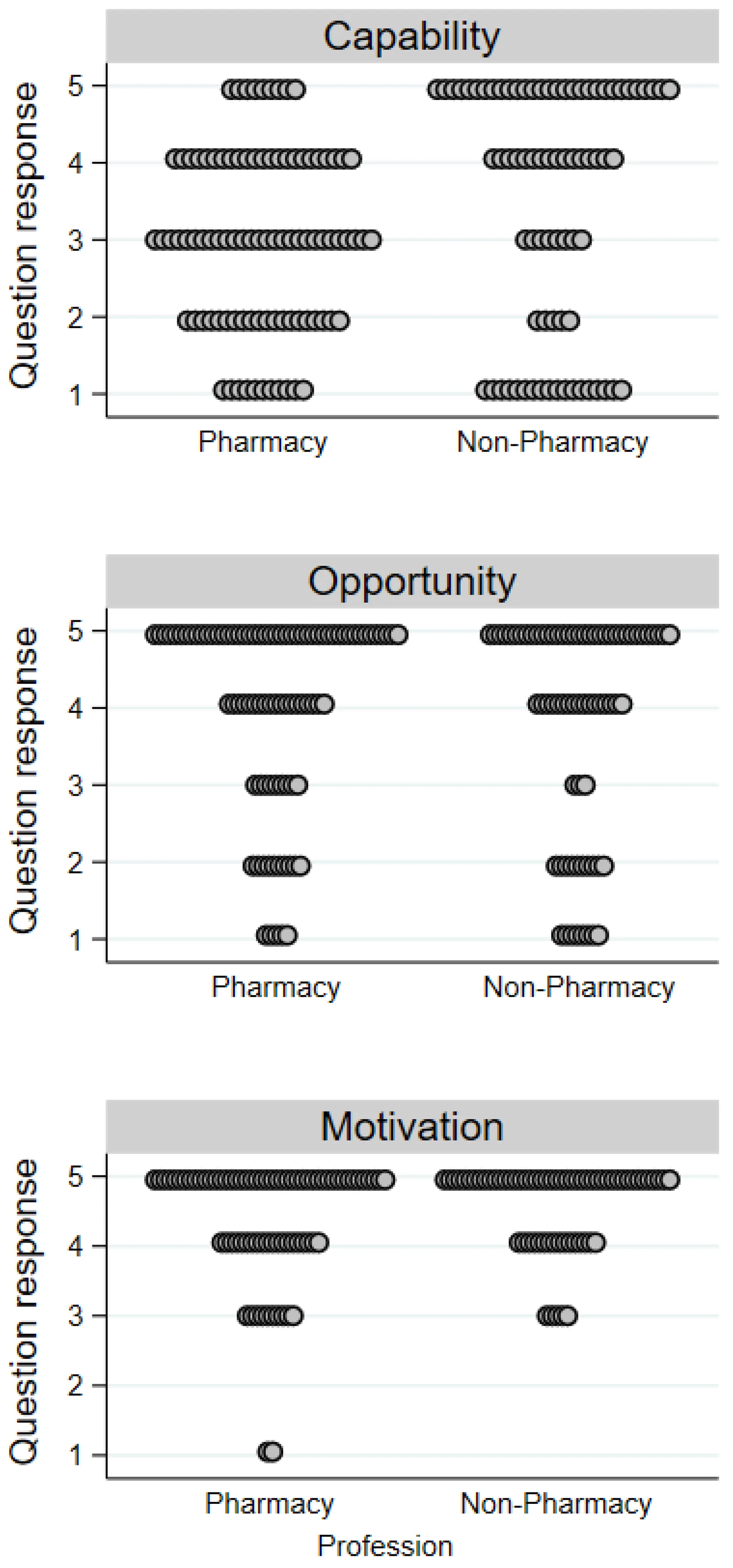
Responses to capability, opportunity and motivation questions, stratified by professional group. All responses were coded on a 5-point Likert scale: 1 = strongly disagree; 2 = slightly disagree; 3 = neither agree nor disagree; 4 = slightly agree; 5 = strongly agree.

The capability, opportunity, and motivation responses for pharmacy and non-pharmacy professionals are displayed in Figure 1.

The responses to the individual capability, opportunity and motivation questions are summarized in Table 2, Table 3 and Table 4.

In general, the findings suggested non-pharmacy professionals had higher self-rated levels of capability than pharmacy professionals with respect to acne prescribing and improving prescribing practices, although both groups’ responses were broadly positive (Table 2). Both groups rated their skills to run searches on their clinical system as the highest rating capability item (median = 5 for both groups). Non-pharmacy professionals also rated being able to give self-care advice to people with acne as equally high. All other questions, except “I am able to give self-care advice to people with acne”, scored joint lowest for pharmacy professionals (median = 3 for all). Understanding the review criteria for a step down of antibiotic treatment for acne had the lowest median score for non-pharmacy professionals (median = 3).

Overall, the responses indicated there were opportunities for both professional groups to tackle AMR and improve prescribing practices, with potentially slightly higher levels of opportunity for pharmacy compared to non-pharmacy professionals (Table 3). Most questions had the joint median highest scores for pharmacy professionals, except for having protected time for training (median = 3), the opportunity to run searches on the clinical system to monitor antibiotic prescribing in the past 3 months (median = 3.5), and support staff to run searches (median = 4).

For non-pharmacy professionals, the highest median scores reflected that AMS and antibiotic prescribing review were priorities in the workplace and that they had easy access to guidelines needed for managing infections (median = 5). The lowest median score suggested limited opportunities to run searches on the clinical system to monitor antibiotic prescribing (median = 1).

However, for those who do not have the opportunity to run clinical searches, 36.4% (4/11) agreed or strongly agreed that they had support staff to help.

Responses to motivation questions suggested high levels of motivation with respect to acne prescribing and AMR (Table 4). Both groups of professionals strongly agreed with all questions (median = 5 for all), except for the statement “The review of patients on treatment for acne gives me job satisfaction” (median = 3 for pharmacy professionals; median = 4 for non-pharmacy professionals).

### 2.2. Antibiotic and AMR Knowledge

Seven key knowledge questions assessed healthcare professionals’ knowledge about antibiotics and AMR. The data showed that 79% of respondents were able to answer all seven questions correctly.

Among pharmacy professionals, one question received some incorrect answers: 79% of respondents correctly identified that “Every person treated with antibiotics is at an increased risk of antibiotic-resistant infection”. Among non-pharmacy professionals, two questions received some incorrect answers: 91% of respondents correctly identified that “Every person treated with antibiotics is at an increased risk of antibiotic-resistant infection”, and 91% of respondents correctly agreed that “Antibiotic-resistant bacteria can spread from person to person”.

### 2.3. Clinical Behavior

Eleven individuals (45.83%) currently prescribed antibiotics; four were pharmacy professionals, and seven were non-pharmacy professionals. Among these people, four prescribed antibiotics daily, four prescribed them weekly and two prescribed them monthly (one individual was not sure). Only three respondents (all doctors) had reviewed one or more patients for treatment of acne in the month prior to responding to the survey.

Table 5 summarizes responses relating to clinical behavior. Fifteen respondents (62.5%) prescribed, dispensed or administered antibiotics once or more in the previous week. Patient information about antibiotic use was infrequently used (20.8% used at least once in the past week), though verbal advice was more common (41.7% provided at least once in the past week).

Four out of eleven (36.4%) prescribers reported prescribing an antibiotic at least once in the previous week when they would have preferred not to. Fears of patient deterioration or complications led 6 out of 11 (54.6%) respondents to prescribe antibiotics in the preceding week and because the respondents were uncertain about the diagnosis (36.4%). Rarely in the previous week were antibiotics prescribed because it was faster than explaining why the patient should not have them (9.1%), or 18.2% of respondents prescribed an antibiotic in the past week to maintain the relationship with the patient.

Three out of eleven (27.3%) respondents discontinued treatment within 3 days during the past week because a bacterial infection was considered unlikely, while two out of eleven (18.2%) reported stopping an antibiotic prescription earlier than the prescribed course length. Only 1 out of 11 (9.1%) respondents reported prescribing a shorter course than recommended in guidelines.

### 2.4. Feedback on the ‘How to…’ Resources

A minority of respondents were aware of the TARGET “Acne How To” toolkit prior to the survey (4/24, 16.7%). This included two pharmacy professionals and two non-pharmacy professionals, none of whom had used the toolkit before.

Of those who were aware of the toolkit or accessed it during the survey, 80% (8/10) felt it was appropriate for health and justice settings, with responses split between pharmacy professionals and non-pharmacy professionals. Two pharmacy professionals did not find it appropriate for health and justice settings.

Similarly, only a minority of respondents had accessed the acne worked example PowerPoint accompanying the toolkit (7/23, 30.4%); among whom, six (85.7%) felt it was applicable to health and justice settings. This comprised three pharmacy professionals and three non-pharmacy professionals.

## 3. Discussion

The aim of this exploratory study was to understand prescribing practices and AMR attitudes, with a secondary focus on acne prescribing, among health and justice healthcare professionals, using the COM-B model. While the survey instrument was based on previously used COM-B questionnaire [10], its application within English correctional facilities addresses an important evidence gap. Healthcare professionals working in these settings operate within a context that differs from most community and hospital services, yet little research has explored their perspectives on AMS, prescribing behavior and antimicrobial resistance.

The survey results suggest that most respondents generally demonstrated a good understanding of AMR, while motivation scores were higher than capability and opportunity scores. Given the small sample size and descriptive nature of this study, these findings should be interpreted cautiously and viewed as areas for future investigation rather than definitive evidence of specific intervention needs.

Should these results be replicated, the Behavior Change Wheel proposes several approaches to support behavior change. This includes education, persuasion, incentivization, training, environmental restructuring, modeling and enablement [11]. Although the current findings may help identify areas where such interventions could be explored in health and justice settings, additional research is required to understand which barriers are most important and which intervention approaches are likely to be feasible and effective.

Several contextual factors within correctional facilities could potentially influence healthcare professionals’ capability, opportunity and motivation with respect to AMS. The structure of the setting, transfers, and the correctional facility regime may all constrain health and justice healthcare staff, resulting in competing health priorities that limit the ability to undertake AMS [12]. Furthermore, individuals in contact with the criminal justice system tend to have more complex health needs compared to the general population, which may lead to a greater demand for antibiotics and more complex considerations when prescribing due to multimorbidity and polypharmacy [13], which could impact opportunity, capability, and motivation to prioritize AMS programs.

Furthermore, the correctional facility’s setting and patient characteristics will also impact upon antibiotic prescribing. First, levels of infections, including acne, may be higher in correctional facilities due to stress, suboptimal hygiene conditions [7], and limited access to skincare products, which may exacerbate acne. Second, incarcerated individuals may be less engaged with community healthcare services [14], so correctional healthcare staff might treat even mild infections and cases of acne, seeing this as an opportunity to address a health issue that incarcerated individuals may otherwise struggle to address after release. Additionally, correctional healthcare staff may face pressures to provide rapid support for people who present with complex, unmet medical needs, especially when they may not be incarcerated for long periods. Prescribing for acne, for example, allows them to address at least one manageable issue, even if more critical problems require longer-term solutions. Finally, continuity of care is complex in correctional facilities. Correctional facilities’ healthcare staff may continue existing prescriptions for newly incarcerated people that they might not have prescribed initially to avoid adding stress by altering a familiar treatment plan during a challenging transition period for the incarcerated individual.

Collectively, this may result in staff in correctional facilities being less able to adopt AMS programs, thus risking inequitable healthcare and widening health inequalities for populations known to have worse health outcomes than those in the community [15].

Despite the unique challenges found in correctional facilities, understanding of AMR was broadly similar to the results of another survey, which posed the same seven key knowledge questions to 18,365 European health professionals [10]. In this study, 79% of respondents were able to answer all seven knowledge questions correctly. This was higher than the EU average of 58%, with the UK scoring 59%. Data from the European questionnaire will be used as a baseline with a 10% improvement in knowledge as a target in the new AMR NAP. A 10% improvement would bring the UK in line with the highest-scoring EU countries. The two statements in this survey that had incorrect responses were also the statements with the lowest percentage of correct responses in the European-wide survey. In the latter survey, 75% of respondents correctly identified that “Every person treated with antibiotics is at an increased risk of antibiotic-resistant infection”, and 86.9% of respondents identified that “Antibiotic-resistant bacteria can spread from person to person”.

When considering these survey findings, it is pertinent to point out the differences in the numbers of respondents (141 respondents for the UK survey, and 18,365 for the European-wide survey, of whom 2404 were from the UK). This may also underline some variations. The small number of respondents to our survey (*n* = 24) means there is a greater degree of statistical uncertainty about these results, which will be more influenced by chance.

Furthermore, the responses for this survey were substantially smaller than the calculated sample size requirement, and the descriptive nature of this study means that it was not designed or powered to formally compare between professional groups. Therefore, observations regarding potential differences between groups or possible explanations for the findings should be viewed as hypothesis-generating.

Convenience sampling was used, and it was not possible to determine a response rate or assess the representativeness of respondents due to the absence of national workforce demographic data for health and justice healthcare staff. Therefore, the findings should be considered exploratory rather than representative of all healthcare professionals working in English correctional facilities.

Several factors may have contributed to the low number of survey responses. Healthcare professionals working in correctional settings frequently operate within resource-constrained environments and manage complex patient populations with significant physical and mental health needs, leaving limited protected time for participation in surveys and research activities. In addition, the survey was distributed through professional networks rather than through a comprehensive national register of correctional healthcare staff, meaning that not all eligible individuals may have received the invitation. It is also possible that respondents were more likely to have an existing interest in AMS, introducing self-selection bias. Collectively, these factors may have affected both participation and the generalizability of the findings.

Despite these acknowledged limitations, there are several strengths to this study. This is the first study, to the authors’ knowledge, that has assessed health and justice healthcare professionals’ knowledge, attitudes, and behaviors towards AMS within English correctional facilities. This is a crucial topic, which contributes to tackling health inequalities experienced by individuals in correctional facilities, and also contributes to the UK government’s 5-year national action plan to tackle AMR [11]. This provides preliminary evidence, indicating where there may be a need for further research.

Although this study does not establish whether healthcare professionals in correctional facilities differ from those in other healthcare settings, it provides baseline evidence from a population and service context that has received little attention in the AMS literature. The findings can be used to inform future research, intervention development and service improvement initiatives tailored to health and justice settings. For example, only 20.8% of respondents gave out resources about prudent antibiotic use or management of infections such as leaflets or pamphlets to patients. It is essential to understand whether this is because they do not have access to them or whether they are available but not useful or appropriate.

Beyond the need for more research in this area, it is important to develop interventions to improve health and justice healthcare professionals’ capability, opportunity and motivation to improve antimicrobial prescribing practices, especially in light of the large health inequalities experienced by the populations they serve [15] and the high levels of acne prescribing and broad-spectrum antibiotic prescribing found nationally across English correctional facilities (paper under review). Of note, a minority of respondents had accessed the TARGET acne ‘How to’ resources [16], suggesting that one potential intervention may be to increase awareness of these resources.

## 4. Materials and Methods

This study is reported in line with the Consensus-Based Checklist for Reporting of Survey Studies (CROSS) guidelines [17]. The CROSS checklist is reported in Table S1.

### 4.1. Study Design and Participants

A quantitative cross-sectional survey was undertaken to understand the prescribing practices, particularly in relation to acne, and attitudes towards AMS among health and justice healthcare professionals. Participants were based in English correctional facilities, which includes facilities used for the detention of young people and adults who are either sentenced by the courts or held on remand. It includes Young Offender Institutions, Secure Training Centers, Secure Children’s Homes and all prisons.

Convenience sampling was used as the survey was shared via professional healthcare networks.

### 4.2. Survey Methodology

The COM-B model was applied to assess the capability, opportunity and motivation of health and justice healthcare professionals. Statements relating to prescribing antibiotics and the management of acne were developed and categorized as assessing ‘capability’, ‘opportunity’ or ‘motivation’. Additional questions relating to clinical behavior, including antibiotic prescribing and acne management, were also included. Except for demographic and consent questions, there were 82 statements based on a previously validated survey [10]. In addition, there were seven key AMR knowledge questions, also based on a previously validated survey [18].

The survey was hosted on an electronic platform (UKHSA’s People Pulse). To reduce non-response bias by improving readability and length, the survey was pretested once by a member of the target population (a pharmacist working in a correctional facility). A mobile-friendly design was also used to increase accessibility, and participation certificates were offered to incentivize participation.

After initial dissemination, three reminders were sent out to prompt eligible people to take part. The survey was live for 3 weeks.

For most questions, participants rated their agreement with the questions on a 5-point Likert scale, which was rated on a 1–5 scale (strongly disagree to strongly agree). The key AMR knowledge questions had True–False responses, open-text fields, or yes/no/don’t know options. There were questions specifically for professionals who prescribed or managed someone with acne in the preceding month and, thus, were not answered by all respondents. Demographic questions (profession, summarized as pharmacy professional or non-pharmacy professional, English region of practice, and duration of time working in health and justice) were asked at the end of the survey.

The full survey is provided in Table S2. The software used could not prevent individuals from completing the survey multiple times. However, assessment of the demographics and responses does not indicate any likely duplications.

Consent to participate was gathered via completion of the survey. The text displayed upon starting the survey and used to gain consent from participants is provided in Table S3.

### 4.3. Data Analysis

The demographic characteristics were summarized by number of respondents and percentage. Responses to Likert-scale questions were treated as ordinal data and summarized using medians and interquartile ranges (IQRs), with 1 equaling strongly disagree and 5 equaling strongly agree, with additional responses of 6 = don’t know/not sure and 7 = not applicable. For one question (“In the last three months I have had the opportunity to run searches on the clinical system to monitor antibiotic prescribing”), the scale was coded: 1 = never, 2 = rarely, 3 = sometimes, 4 = often, and 5 = frequently. Given the exploratory nature of this study and the small sample size, analyses were descriptive.

No survey questions had missing data, although not all survey questions were mandatory, so the number of responses varied. For questions relating specifically to acne, one respondent’s answers were excluded because their profession meant that they would not be responsible for acne treatment.

Results were also summarized separately for pharmacy professionals (including pharmacists and pharmacy technicians) and non-pharmacy professionals. The division into two professional groups (pharmacy professionals, non-pharmacy professionals) was chosen based on their roles in relation to AMS. For example, the high level of involvement of pharmacy staff in implementing AMS strategies in primary and secondary care suggests different levels of capability, opportunity, motivation and/or behavior.

Formal tests were not conducted due to the limited number of respondents, which limited statistical power and increased the risk of Type 2 errors. Prior to survey distribution, a sample size calculation was undertaken using a significance level of 0.05, statistical power of 0.8, standard deviation of 0.468 [10], and a conservative effect size of 0.09 [10]. This indicated a required sample size of 848. The sample size achieved was substantially lower (*n* = 24). Therefore, this study was considered exploratory and descriptive in nature, and formal inferential statistical analyses between pharmacists and non-pharmacists were not undertaken.

Due to a lack of information about non-respondents and no publicly available demographic data about healthcare staff working in correctional facilities, it was not possible to adjust for any potential non-representativeness of the sample or non-response error.

It was not possible to calculate the response rate as the survey was shared within professional networks, and it is unclear how many individuals it reached.

Data were analyzed using MS Excel (2010) and STATA statistical software release 15 (StataCorp, College Station, TX, USA).

### 4.4. Ethical Statement

Ethical considerations were assessed using the NHS Health Research Authority guidance. The assessment determined that NHS Research Ethics Committee review was not required. This was because the study involved healthcare professionals participating solely in their professional capacity and did not involve NHS patients or service users, offenders as research participants, collection of human tissue, access to confidential patient information, or any other category requiring review by an NHS Research Ethics Committee under HRA guidance. Although the study concerned healthcare delivery within correctional facilities, no incarcerated individuals were recruited or participated in the research; all participants were healthcare professionals acting in their professional roles. A copy of the HRA decision tool output was retained by the research team.

All respondents participated strictly in their professional capacity and were provided with participant information and informed consent prior to participation, according to the Declaration of Helsinki.

No personally identifiable information was collected through the survey so responses cannot be traced back to respondents. To receive a certificate of completion or express further interest in the work, a separate form was completed, so that personal details were kept separate from survey responses. Survey data were securely stored, and only authorized personnel could access raw survey data.

The views expressed in this study are those of the author(s) and are not necessarily those of UK Health Security Agency or NHS England.

## 5. Conclusions

This exploratory study suggests that participating health and justice healthcare professionals have a strong understanding of AMR and generally positive motivation towards AMS. However, capability- and opportunity-related responses identified potential areas for improvement. Given the small sample size and non-probability sampling approach, these findings should be interpreted cautiously and warrant confirmation in larger studies. The findings provide preliminary insights into AMS within an under-researched setting and may help inform future research and intervention development.

## Figures and Tables

**Table 1:** Demographic characteristics of respondents.

		Pharmacy Professionals		Non-Pharmacy Professionals	
		*N*	%	*N*	%
Duration of time as a healthcare professional in the UK	0–5 years	0	0	1	9.09
	6–10 years	4	30.77	0	0
	11–15 years	2	15.38	4	36.36
	16–20 years	5	38.46	0	0
	More than 20 years	2	15.38	6	54.55
Current region of practice	East of England	3	23.08	2	18.18
	London	1	7.69	3	27.27
	Midlands	2	15.38	0	0
	North East and Yorkshire	1	7.69	1	9.09
	North West	1	7.69	0	0
	South East	5	38.46	4	36.36
	South West	0	0	1	9.09
Gender	Female	8	61.54	7	63.64
	Male	5	38.46	4	36.36

**Table 2:** Median 5-point Likert responses to capability questions in the survey, by professional group (*n* = 24).

	Pharmacy Professionals		Non-Pharmacy Professionals		Total	
	Median	IQR	Median	IQR	Median	IQR
I have the skills to run searches on my clinical system	5	1	5	2	5	1
I have enough knowledge to manage people with acne	3	1	4	2	4	1
I am able to give self-care advice to people with acne	4	2	5	1	4	3
I understand when onward referral is needed for acne	3	1	4.5	3	3	2
I have enough knowledge to undertake reviews with patients with repeated or long-term use of antibiotics for acne management	3	2	4	2	3	2
I understand the review criteria for stepping up treatment for acne	3	1	4	3	3	2
I understand the review criteria for a step down of antibiotic treatment for acne	3	1	3.5	3	3	2

Responses were coded on a 5-point Likert scale: 1 = strongly disagree; 2 = slightly disagree; 3 = neither agree nor disagree; 4 = slightly agree; 5 = strongly agree. Half-point values (e.g., 3.5 and 4.5) reflect median scores lying between two adjacent response categories.

**Table 3:** Median 5-point Likert responses to opportunity questions in the survey, by professional group (*n* = 24).

	Pharmacy Professionals		Non-Pharmacy Professionals		Total	
	Median	IQR	Median	IQR	Median	IQR
I have easy access to guidelines I need for managing infection	5	0	5	0	5	0
I have easy access to the materials I need to give advice on prudent antibiotic use and antibiotic resistance	5	1	4	3	5	1
I have good opportunities to provide advice on prudent antibiotic use to individuals	5	1	5	3	5	1.5
Antimicrobial stewardship and antibiotic prescribing review is a priority in my workplace	5	2	4.5	1	5	2
I use protected time for training when new toolkits or training materials relevant to my job are issued	3	2	4	3	4	2
I am able to undertake quality improvement initiatives on areas of medicines use OR prescribing that I have an interest in	5	1	4	2	4	2
There are support staff in my practice to run searches about antibiotics on my behalf	4	2.5	3	4	4	3
In the last three months I have had the opportunity to run searches on the clinical system to monitor antibiotic prescribing *	3.5	3	1	2	2.5	4

Unless otherwise indicated, responses were coded on a 5-point Likert scale: 1 = strongly disagree, 2 = slightly disagree, 3 = neither agree nor disagree, 4 = slightly agree, and 5 = strongly agree. Half-point values (e.g., 2.5, 3.5 and 4.5) reflect median scores lying between two adjacent response categories. Items marked with an asterisk (*) were coded on a frequency scale: 1 = never, 2 = rarely, 3 = sometimes, 4 = often, and 5 = frequently. Higher scores indicate greater perceived opportunity.

**Table 4:** Median 5-point Likert responses to motivation questions in the survey, by professional group (*n* = 24).

	Pharmacy Professionals		Non-Pharmacy Professionals		Total	
	Median	IQR	Median	IQR	Median	IQR
Managing acne appropriately is important for the patient’s quality of life	5	0	5	0	5	0
Managing the prescribing of antibiotics for acne appropriately can impact on antibiotic resistance	5	1	5	0	5	0
Appropriate self-care advice is important to avoid unnecessary antibiotic use for acne	5	1	5	0	5	0
I know there is a connection between my prescribing OR dispensing OR administering of antibiotics and emergence and spread of antibiotic-resistant bacteria	5	1	5	1	5	1
I have a key role in helping control antibiotic resistance	5	1	5	0	5	1
The review of patients on treatment for acne gives me job satisfaction	3	1	4	2	4	1

All responses were coded on a 5-point Likert scale: 1 = strongly disagree; 2 = slightly disagree; 3 = neither agree nor disagree; 4 = slightly agree; 5 = strongly agree.

**Table 5:** Number of individuals with concerns that impact antibiotic prescribing behavior in the week prior to completing the survey, by professional group.

		Pharmacy Professionals		Non-Pharmacy Professionals		Total	
How frequently did you prescribe/dispense (clinically screened prescription)/administer antibiotics?	Less than once a week, never or don’t know	7	53.9%	2	18.2%	9	37.5%
	Once a week or more frequently	6	46.2%	9	81.8%	15	62.5%
How frequently did you give out resources (e.g., leaflets or pamphlets) on prudent antibiotic use or management of infections to individuals?	Less than once a week, never or don’t know	10	76.9%	9	81.8%	19	79.2%
	Once a week or more frequently	3	23.1%	2	18.2%	5	20.8%
How frequently did you give out advice related to prudent antibiotic use or management of infections to individuals?	Less than once a week, never or don’t know	8	61.5%	6	54.6%	14	58.3%
	Once a week or more frequently	5	38.5%	5	45.5%	10	41.7%
How often would you have preferred not to prescribe an antibiotic but had to? *	Less than once a week, never or don’t know	3	75.0%	4	57.1%	7	63.6%
	Once a week or more frequently	1	25.0%	3	42.9%	4	36.4%
How often did the fear of patient deterioration or fear of complications lead you to prescribe antibiotics? *	Less than once a week, never or don’t know	2	50.0%	5	71.4%	7	63.6%
	Once a week or more frequently	2	50.0%	4	57.1%	6	54.6%
How often did you prescribe antibiotics because it took less time than to explain the reason why they are not indicated? *	Less than once a week, never or don’t know	4	100.0%	6	85.7%	10	90.9%
	Once a week or more frequently	0	0.0%	1	14.3%	1	9.1%
How often did you prescribe an antibiotic because you were uncertain about the diagnosis of infection? *	Less than once a week, never or don’t know	2	50.0%	5	71.4%	7	63.6%
	Once a week or more frequently	2	50.0%	2	28.6%	4	36.4%
How often did you prescribe an antibiotic to maintain the relationship with the patient? *	Less than once a week, never or don’t know	3	75.0%	6	85.7%	9	81.8%
	Once a week or more frequently	1	25.0%	1	14.3%	2	18.2%
How often did you stop an antibiotic prescription earlier than the prescribed course length? *	Less than once a week, never or don’t know	2	50.0%	7	100.0%	9	81.8%
	Once a week or more frequently	2	50.0%	0	0.0%	2	18.2%
How often did you discontinue early (within three days after initiation) a treatment because bacterial infection was not likely after all? *	Less than once a week, never or don’t know	2	50.0%	6	85.7%	8	72.7%
	Once a week or more frequently	2	50.0%	1	14.3%	3	27.3%
How often did you prescribe a shorter course of treatment as compared to available guidelines? *	Less than once a week, never or don’t know	3	75.0%	7	100.0%	10	90.9%
	Once a week or more frequently	1	25.0%	0	0.0%	1	9.1%

* 11 respondents for these questions.

## Data Availability

The original contributions presented in this study are included in the article/Supplementary Material. Further inquiries can be directed to the corresponding author.

## Supplementary Materials

The following supporting information can be downloaded at: https://www.mdpi.com/article/10.3390/antibiotics15090911/s1, Table S1: Checklist for Reporting of Survey Studies (CROSS); Table S2: Full list of survey questions shared with health and justice healthcare workers in English correctional facilities, 2024; Table S3: Introductory text used to explain the survey and gain consent.

## Abbreviations

The following abbreviations are used in this manuscript:

AMR	Antimicrobial Resistance
NHS	National Health Service
AMS	Antimicrobial Stewardship
COM-B	Capability, Opportunity, Motivation and Behavior Model
CQUIN	Commissioning for Quality and Innovation
CROSS	Consensus-Based Checklist for Reporting of Survey Studies
UKHSA	UK Health Security Agency
