# Supplementary material for: Exploring Healthcare Workers’ Knowledge, Attitudes and Behaviors to Antibiotic Use, Including in Acne, and Antibiotic Resistance in English Correctional Facilities in 2024
Source: Antibiotics (Basel). 2026 Sep 16;15(9):911. doi: 10.3390/antibiotics15090911 (PMC13603517; doi:10.3390/antibiotics15090911)
Supplement: Supplementary file 1 [file antibiotics-15-00911-s001.zip › antibiotics-4478504-supplementary.pdf]

Supplementary Materials

Table S1. Checklist for Reporting of Survey Studies (CROSS).

Section /topic	Item	Item description	Reported on page #
**Title and abstract**			
**Title and abstract**	1a	State the word "survey" along with a commonly used term in title or abstract to introduce the study's design.	2
	1b	Provide an informative summary in the abstract, covering background, objectives, methods, findings/results, interpretation/discussion, and conclusions.	2
**Introduction**			
**Background**	2	Provide a background about the rationale of study, what has been previously done, and why this survey is needed.	3
**Purpose/aim**	3	Identify specific purposes, aims, goals, or objectives of the study.	3
**Methods**			
**Study design**	4	Specify the study design in the methods section with a commonly used term (e.g., cross-sectional or longitudinal).	4
**Data collection methods**	5a	Describe the questionnaire (e.g., number of sections, number of questions, number and names of instruments used).	4
	5b	Describe all questionnaire instruments that were used in the survey to measure particular concepts. Report target population, reported validity and reliability information, scoring/classification procedure, and reference links (if any).	4
	5c	Provide information on pretesting of the questionnaire, if performed (in the article or in an online supplement). Report the method of pretesting, number of times questionnaire was pre-tested, number and demographics of participants used for pretesting, and the level of similarity of demographics between pre-testing participants and sample population.	4
	5d	Questionnaire, if possible, should be fully provided (in the article, or as appendices or as an online supplement).	eTable 2
**Sample characteristics**	6a	Describe the study population (i.e., background, locations, eligibility criteria for participant inclusion in survey, exclusion criteria).	4
	6b	Describe the sampling techniques used (e.g., single stage or multistage sampling, simple random sampling, stratified sampling, cluster sampling, convenience sampling). Specify the locations of	4

		sample participants whenever clustered sampling was applied.	
	6c	Provide information on sample size, along with details of sample size calculation.	4
	6d	Describe how representative the sample is of the study population (or target population if possible), particularly for population-based surveys.	5-6
**Survey administration**	7a	Provide information on modes of questionnaire administration, including the type and number of contacts, the location where the survey was conducted (e.g., outpatient room or by use of online tools, such as SurveyMonkey).	4
	7b	Provide information of survey's time frame, such as periods of recruitment, exposure, and follow-up days.	4
	7c	Provide information on the entry process: For web-based surveys, provide approaches to prevent "multiple participation" of participants.	4
**Study preparation**	8	Describe any preparation process before conducting the survey (e.g., interviewers' training process, advertising the survey).	4
**Ethical considerations**	9a	Provide information on ethical approval for the survey if obtained, including informed consent, institutional review board [IRB] approval, Helsinki declaration, and good clinical practice [GCP] declaration (as appropriate).	5
	9b	Provide information about survey anonymity and confidentiality and describe what mechanisms were used to protect unauthorized access.	eTable 3, 5
**Statistical analysis**	10a	Describe statistical methods and analytical approach. Report the statistical software that was used for data analysis.	5
	10b	Report any modification of variables used in the analysis, along with reference (if available).	n/a
	10c	Report details about how missing data was handled. Include rate of missing items, missing data mechanism (i.e., missing completely at random [MCAR], missing at random [MAR] or missing not at random [MNAR]) and methods used to deal with missing data (e.g., multiple imputation).	5
	10d	State how non-response error was addressed.	5
	10e	For longitudinal surveys, state how loss to follow-up was addressed.	n/a
	10f	Indicate whether any methods such as weighting of items or propensity scores have been used to adjust for non-representativeness of the sample.	5
	10g	Describe any sensitivity analysis conducted.	n/a

**Results**			
**Respondent characteristics**	11a	Report numbers of individuals at each stage of the study. Consider using a flow diagram, if possible.	6
	11b	Provide reasons for non-participation at each stage, if possible.	n/a
	11c	Report response rate, present the definition of response rate or the formula used to calculate response rate.	6
	11d	Provide information to define how unique visitors are determined. Report number of unique visitors along with relevant proportions (e.g., view proportion, participation proportion, completion proportion).	4, 6
**Descriptive results**	12	Provide characteristics of study participants, as well as information on potential confounders and assessed outcomes.	6
**Main findings**	13a	Give unadjusted estimates and, if applicable, confounder-adjusted estimates along with 95% confidence intervals and p-values.	6-8
	13b	For multivariable analysis, provide information on the model building process, model fit statistics, and model assumptions (as appropriate).	n/a
	13c	Provide details about any sensitivity analysis performed. If there is a considerable amount of missing data, report sensitivity analyses comparing the results of complete cases with that of the imputed dataset (if possible).	n/a
**Discussion**			
**Limitations**	14	Discuss the limitations of the study, considering sources of potential biases and imprecisions, such as non-representativeness of sample, study design, important uncontrolled confounders.	10
**Interpretations**	15	Give a cautious overall interpretation of results, based on potential biases and imprecisions and suggest areas for future research.	9
**Generalizability**	16	Discuss the external validity of the results.	9-10
**Other sections**			
**Role of funding source**	17	State whether any funding organization has had any roles in the survey's design, implementation, and analysis.	
**Conflict of interest**	18	Declare any potential conflict of interest.	
**Acknowledgements**	19	Provide names of organizations/persons that are acknowledged along with their contribution to the research.	1

Table S**2.** Full list of survey questions shared with health and justice healthcare workers in English correctional facilities, 2024.

Question	Response Options
**1) Please select your professional group**	Pharmacist<br>Pharmacy technician,<br>Nurse<br>Doctor<br>Other
**2) In the last 12 months, do you remember receiving any information about avoiding unnecessary prescribing OR administering OR dispensing of antibiotics?**	Yes/No/Not Sure
**3) On the basis of the information you received, have you changed your practice on prescribing OR administering OR dispensing of antibiotics?**	Yes/No/Not Sure
**4) If yes, please list what has had the most influence on changing your practice?**	Open-text Field
**5) If no, why not?**	Open-text Field
**6) How effective do you believe the following campaigns have been in raising awareness about prudent use of antibiotics and antibiotic resistance?**	5-point Likert scale: scale of 1 to 5, where 1 is "very ineffective" and 5 is "very effective"
**European Antibiotic Awareness Day (EAAD)**	
**World Antibiotic Awareness Week (WAAW)**	
**Treat Antibiotics Responsibly, Guidance, Education Tools (TARGET Antibiotics tools)**	
**Antibiotic Guardian**	
**Keep Antibiotics Working**	
**eBug**	
**Start Smart then Focus (SSTF)**	
**NICE guidelines**	
**SIGN guidelines**	
**7) What resources have you accessed in the past 12 months to improve your knowledge of antimicrobial resistance and infection management (select all that apply)**	Selection of options:<br>1.UKHSA Target resources hosted by the Royal College of GPs (RCGP)<br>a.Diagnostic tools e.g. UTI<br>b.Audit templates e.g. UTI or cough<br>c.Training resources e.g. managing UTI eModule or webinar<br>d. Other TARGET resources<br>2. British Society for Antimicrobial Chemotherapy (BSAC)<br>3 Electronic learning for health (e-lfh)<br>4. Centre for Postgraduate Pharmacy Education (CPPE)<br>5. PrescQipp<br>6.United Kingdom Clinical Pharmacy Association (UKCPA)<br>7.Other (please state)

**8) Were you aware of the TARGET "how to…" resource for acne management prior to this survey**	Yes, and I have used it<br>Yes, but I have not used it<br>No
**Capability**	
**9) Please answer whether you believe these statements are true or false.**<br>**a. Antibiotics are effective against viruses**<br>**b. Antibiotics are effective against cold and flu**<br>**c. Unnecessary use of antibiotics increases the risk of them becoming ineffective**<br>**d. Taking antibiotics has associated side effects or risks such as diarrhoea, nausea, colitis, allergies and thrush**<br>**e. Every person treated with antibiotics is at an increased risk of antibiotic-resistant infection**<br>**f. Antibiotic-resistant bacteria can spread from person to person**<br>**g. Healthy people can carry antibiotic-resistant bacteria**	True/False
**10) I have the skills to run searches on my clinical system**<br>**11) To what extent do you agree or disagree with the following statements:**<br>**I have enough knowledge to manage people with acne**<br>**12) I am able to give self-care advice to people with acne**<br>**13) I have enough knowledge to undertake reviews with patients with repeated or long-term use of antibiotics for acne management**<br>**14) I understand the review criteria for stepping up treatment for acne**<br>**15) I understand the review criteria for a step down of antibiotic treatment for acne**<br>**16) I understand when onward referral is needed for acne**	5-point Likert (1 = strongly disagree, 2 = slightly disagree, 3 = neither agree or disagree, 4 = slightly agree, 5 = strongly agree)
**Opportunity**	
**17) To what extent do you agree or disagree with the following statements?**<br>**- I have easy access to guidelines I need on managing infections**<br>**- I have easy access to risk assessment tools (such as clinical scores) to target antibiotics to patients who are most likely to benefit from them**<br>**- I have easy access to point-of-care diagnostic tests to target antibiotics to patients who are most likely to benefit from them**	5-point Likert (1 = strongly disagree, 2 = slightly disagree, 3 = neither agree or disagree, 4 = slightly agree, 5 = strongly agree); don't know/not sure; N/A

**- I have easy access to the materials I need to give advice on prudent antibiotic use and antibiotic resistance**	
**- I have easy access to shared decision-making tools to use with patients to facilitate informed decision-making on antibiotic treatment**	
**- I have good opportunities to provide advice on prudent antibiotic use to individuals**	
**18) And thinking only about the last week in your clinical practice, how frequently did the statements below apply to you? Please select one option per row. If a question is not applicable then please choose N/A**	5-point Likert (1 = strongly disagree, 2 = slightly disagree, 3 = neither agree nor disagree, 4 = slightly agree, 5 = strongly agree); don't know/not sure; N/A
**- Prescribed / dispensed (clinically screen prescription) / administered antibiotics**	
**- Gave out resources (e.g. leaflets or pamphlets) on prudent antibiotic use or management of infections to individuals**	
**- Gave out advice related to prudent antibiotic use or management of infections to individuals**	
**19) In the last three months I have had the opportunity to run searches on the clinical system to monitor antibiotic prescribing**	5-point Likert (1 = strongly disagree, 2 = slightly disagree, 3 = neither agree nor disagree, 4 = slightly agree, 5 = strongly agree); don't know/not sure; N/A
**20) There are support staff in my practice to run searches about antibiotics on my behalf**	
**21) Antimicrobial stewardship and antibiotic prescribing review is a priority in my workplace**	
**22) I am able to undertake quality improvement initiatives on areas of medicines use OR prescribing that I have an interest in**	
**23) I use protected time for training when new toolkits or training materials relevant to my job are issued**	
**Motivation**	
**24) To what extent do you agree or disagree with the following statement?**	5-point Likert (1 = strongly disagree, 2 = slightly disagree, 3 = neither agree nor disagree, 4 = slightly agree, 5 = strongly agree)
**- I know there is a connection between my prescribing OR dispensing OR administering of antibiotics and emergence and spread of antibiotic-resistant bacteria**	
**- I have a key role in helping control antibiotic resistance**	
**- Appropriate self-care advice is important to avoid unnecessary antibiotic use for acne**	
**- Managing the prescribing of antibiotics for acne appropriately can impact on antibiotic resistance**	

**- Managing acne appropriately is important for the patient's quality of life**<br>**- The review of patients on treatment for acne gives me job satisfaction**	
**Acne management activity**	
**25) Have you reviewed any patients on treatment for acne in the last month?**<br><br>**If yes go to Q26**<br>**If no go to Q29**	Yes/No/Can't remember
**26) Have you reviewed any patients on antibiotic treatment for acne in the last month?**<br><br>**If yes go to Q27**<br>**If no go to Q29**	Yes/No/Can't remember
**27) In the last month, have you:**<br>**a. stepped any patients down from antibiotic therapy?**<br>**b. referred any patients to dermatology?**<br>**c. referred any patients with acne for mental health support because of their acne?**<br>**d. seen patients but made no change to their long-term treatment?**<br>**e. stepped patients up to antibiotic therapy?**<br>**f. Given advice on self-care for acne**<br>**g. Taken other actions relevant to acne management not specified above (please state)**	Yes/No/can't recall for each statement
**28) When you review patients with acne, to what extent do you use the acne 'how to' guide?**	5-point Likert (1 = strongly disagree, 2 = slightly disagree, 3 = neither agree nor disagree, 4 = slightly agree, 5 = strongly agree); don't know/not sure; N/A
**29) Do you feel that the 'Acne How To' toolkit is applicable to a H&J setting?**	Yes/No/Haven't accessed it
**30) If not, please explain why**	Open-text field
**31) If you have accessed the acne worked example PowerPoint, do you feel that the worked examples are applicable to a H&J setting?**	Yes/No/Haven't accessed it
**32) If not, please explain why**	Open-text field
**Prescriber enablers and barriers to AMS**	

**33) Do you currently prescribe antibiotics?<br>If yes, go to the next question<br>If no, go to Q40**	Yes / No
**34) If yes, how often do you prescribe antibiotics?**	5-point frequency scale: Everyday, Weekly, Monthly, Quarterly, Yearly, don't know / not sure
**35) To what extent do you agree or disagree with the following statement?<br>I am confident making antibiotic prescribing decisions<br>I have confidence in the antibiotic guidelines available to me<br>I have a key role in helping control antibiotic resistance<br>I consider antibiotic resistance when treating a patient<br>I feel supported to not prescribe antibiotics when they are not necessary**	5-point Likert (1 = strongly disagree, 2 = slightly disagree, 3 = neither agree nor disagree, 4 = slightly agree, 5 = strongly agree); don't know / not sure; not applicable
**36) Considering the last one week only:<br>How often would you have preferred not to prescribe an antibiotic but had to during the last one week?<br>How often did the fear of patient deterioration or fear of complications lead you to prescribe antibiotics during the last one week?<br>How often did you prescribe antibiotics because it took less time than to explain the reason why they are not indicated during the last one week?<br>How often did you stop an antibiotic prescription earlier than the prescribed course length during the last one week?<br>How often did you prescribe an antibiotic to maintain the relationship with the patient during the last one week?<br>How often did you prescribe an antibiotic because you were uncertain about the diagnosis of infection during the last one week?<br>How often did you prescribe a shorter course of treatment as compared to available guidelines during the last one week?<br>How often did you discontinue early (within three days after initiation) a treatment because bacterial infection was not likely after all during the last one week?**	6-point frequency scale: 1 = more than once a day, 2 = once a day, 3 = 2-3 times a week, 4 = once a week, 5 = less often than once a week, 6 = never, don't know / not sure

**37) If you are interested in ANY of the following click here**<br>**- A certificate for completing the questionnaire**<br>**- Joining a community of practice for antimicrobial stewardship**<br>**- Helping to tailor the TARGET "how to…" resources for acne for the Health and Justice setting.**<br><br>**By opting in you are under no obligation, but you may be contacted for your views. Please click on the link here to opt in to one or more of the above options.**	Yes/No for each of the three options
**Demographic questions**	
**38) How long have you been a healthcare professional in the UK?**	0-5 years<br>6-10 years<br>11-15 years<br>16-20 years<br>More than 20 years
**39) Where is your current region of practice?**	East of England<br>London<br>East Midlands<br>West Midlands<br>North East and Yorkshire<br>North West<br>South East<br>South West<br>None of the above
**40) What gender do you most identify with?**	Male<br>Female<br>Other identity not listed above<br>Prefer not to say

Table S**3.** Introductory text used to explain the survey and gain consent.

Knowledge, practice and attitudes towards antibiotics and antibiotic resistance.
Thank you for expressing interest in this survey on antimicrobial stewardship!
Before you decide whether you would like to participate, please read the following information, and contact [EMAIL REDACTED] if anything is unclear, or if you would like more information.

What is the purpose of this study?
This study aims to ascertain healthcare workers' antimicrobial resistance knowledge and antimicrobial stewardship behaviours in the health and justice setting. We are also seeking to find out what resources are commonly used for education and training, particularly for acne prescribing.

Who is doing the study?
This survey is being conducted on behalf of the UK Health Security Agency and NHS England. The results will be shared with national stakeholders focussed on tackling antimicrobial resistance in England and inform a programme of national work to improve practice.
Why have I been asked to participate?
You are a clinician working in health and justice settings in England.
Do I have to take part?
No. Taking part is entirely voluntary, and you will be asked to sign a consent form before completing the survey.
What will be involved if I take part in this study?
If you choose to take part, you will complete a survey asking questions related to knowledge, practice and attitudes to antibiotics and antibiotic resistance, especially in relation to acne. It should take no more than 20 minutes to complete. Your input will help us identify barriers to effective antimicrobial stewardship and how to improve antimicrobial stewardship strategies. You may have heard about this survey already via multiple routes; you only need to complete this once so if you have already completed it, please do not proceed any further.
What are the benefits of taking part?
By taking part in the study, you will have the opportunity to have the results of the analysis shared with you, join a community of practice and receive a certificate. We will also provide updates on any subsequent actions or initiatives arising from this survey.
What do I do if I am interested?
Please carefully read the rest of this information sheet. You will then be asked to complete a consent form asking whether or not you voluntarily agree to take part in the study based on the information provided in this participant information sheet. If you agree, you will then be directed to the survey, which should take no more than 20 minutes to complete. Please complete the survey no later than the 30th of June 2024.
Can I withdraw from the study at any time?
You can withdraw from the study at any time (without giving a reason). If you withdraw part way through the survey, any data that you have provided up until that point will not be included in the final dataset.
Will the information obtained in the study be confidential?
No identifiable information is collected as part of the study; this means you will not be identifiable by any of the responses that you provide.
How will the data be stored and what will the data be used for?
All the research we undertake will be GDPR compliant and will abide by the Market Research Society (MRS) Code of Conduct.
Individual responses will be anonymised, and data will be reported in aggregate to maintain anonymity. The results of the survey may be published in anonymised format and made available in the public domain.
Who will have access to my data?
All data are strictly confidential and will be available only to UKHSA and NHS members of staff working on this project and our survey platform provider FlexMR.
How long will the data be retained?
Anonymised data collected through the survey will be stored on secure UKHSA servers and retained for a minimum of 20 years in line with UKHSA policy.
What will happen to the results of the study?
The results of the survey may be published in anonymised format and made available in the public domain.
Who do I contact if I have a concern about the study or I wish to complain?
If you have a concern about any aspect of this study, please speak to naomifleming@nhs.net and we will do our best to answer your query. We will acknowledge your concern within 10 working

days and give you an indication of how it will be dealt with. If you remain unhappy or wish to make a formal complaint, please contact [EMAIL REDACTED].
If you would like more information or have any questions or concerns about the study, please contact [EMAIL REDACTED]. If you encounter any technical difficulties, please contact the survey platform provider FlexMR at [EMAIL REDACTED].
Thank you for taking the time to read this information sheet. Please continue to complete the consent form.

Please read the below bullet points and tick each box to signal agreement with the statements. If you do not select 'I agree' for each one of the statements, please do not participate in the survey.
Please read and respond to each statement below.

	Response
I have read and understood the participant information sheet above.	Agree/Disagree
I understand what my role will be, and my questions have been answered to my satisfaction.	Agree/Disagree
I understand that my participation in the survey is voluntary and that I am free to withdraw at any time, without giving a reason.	Agree/Disagree
I understand I can ask any questions at any time before and during the study.	Agree/Disagree
I understand what will happen to the data collected from me for the study. I consent to the processing of my personal information held by the survey platform provider FlexMR for the purpose of contacting me as part of this study.	Agree/Disagree
I voluntarily agree to take part in this study.	Agree/Disagree

By clicking next you will start the survey and will be providing consent.
